# Endothelin type A receptor blockade attenuates aorto-caval fistula-induced heart failure in rats with angiotensin II-dependent hypertension

**DOI:** 10.1097/HJH.0000000000003307

**Published:** 2022-10-07

**Authors:** Petr Kala, Olga Gawrys, Matúš Miklovič, Zdenka Vaňourková, Petra Škaroupková, Šárka Jíchová, Janusz Sadowski, Elzbieta Kompanowska-Jezierska, Agnieszka Walkowska, Josef Veselka, Miloš Táborský, Hana Maxová, Ivana Vaněčková, Luděk Červenka

**Affiliations:** a Center for Experimental Medicine, Institute for Clinical and Experimental Medicine; b Department of Cardiology, University Hospital Motol and 2nd Faculty of Medicine, Charles University, Prague, Czech Republic; c Department of Renal and Body Fluid Physiology, Mossakowski Medical Research Institute, Polish Academy of Science, Warsaw, Poland; d Department of Internal Medicine I, Cardiology, University Hospital Olomouc and Palacký University, Olomouc; e Department of Pathophysiology, 2nd Faculty of Medicine, Charles University; f Institute of Physiology of the Czech Academy of Sciences, Prague, Czech Republic

**Keywords:** endothelin system, hypertension, Ren-2 renin transgenic rat, renin–angiotensin system, volume-overload heart failure

## Abstract

Evaluation of the effect of endothelin type A (ET_A_) receptor blockade on the course of volume-overload heart failure in rats with angiotensin II-dependent hypertension.

**Methods:**

Ren-2 renin transgenic rats (TGR) were used as a model of hypertension. Heart failure was induced by creating an aorto-caval fistula (ACF). Selective ET_A_ receptor blockade was achieved by atrasentan. For comparison, other rat groups received trandolapril, an angiotensin-converting enzyme inhibitor (ACEi). Animals first underwent ACF creation and 2 weeks later the treatment with atrasentan or trandolapril, alone or combined, was applied; the follow-up period was 20 weeks.

**Results:**

Eighteen days after creating ACF, untreated TGR began to die, and none was alive by day 79. Both atrasentan and trandolapril treatment improved the survival rate, ultimately to 56% (18 of 31 animals) and 69% (22 of 32 animals), respectively. Combined ACEi and ET_A_ receptor blockade improved the final survival rate to 52% (17 of 33 animals). The effects of the three treatment regimens on the survival rate did not significantly differ. All three treatment regimens suppressed the development of cardiac hypertrophy and lung congestion, decreased left ventricle (LV) end-diastolic volume and LV end-diastolic pressure, and improved LV systolic contractility in ACF TGR as compared with their untreated counterparts.

**Conclusion:**

The treatment with ET_A_ receptor antagonist delays the onset of decompensation of volume-overload heart failure and improves the survival rate in hypertensive TGR with ACF-induced heart failure. However, the addition of ET_A_ receptor blockade did not enhance the beneficial effects beyond those obtained with standard treatment with ACEi alone.

## INTRODUCTION

Over the past 40 years, substantial progress has been made in the treatment of acute coronary syndromes. However, many surviving patients still develop substantial myocardial damage eventually leading to heart failure [1]. Heart failure has become a major public health problem [2,3]; despite the availability of multiple therapeutic measures and recent pharmacological advances, the prognosis remains bleak [2,4–7]. Inappropriately activated renin–angiotensin–aldosterone system (RAAS) is crucial for the progression of heart failure and blockade thereof has become a cornerstone component of the treatment. However, in the advanced phase of heart failure its effectiveness is limited [2,6–9], which was conspicuous in patients who had been hypertensive before the onset of heart failure [10–12]. Remarkably, in heart failure induced by volume overload, RAAS inhibition did not attenuate eccentric remodeling of the left ventricle (LV) or improve its systolic function [13–15]. Therefore, new therapeutic strategies for the treatment of heart failure are urgently needed and should be preceded by focused experimental studies [6,16].

It has long been proposed that persistent inappropriate activation of various neurohormonal systems underlies the progression of heart failure (‘neurohormonal model of heart failure pathophysiology’ [17–19]). More recently considerable attention was focused on the endothelin system and its most important peptide: endothelin-1 (ET-1) [20]. ET-1 via endothelin type A (ET_A_) receptors induces vasoconstriction; activation of endothelin type B receptors leads to vasodilatation and natriuresis. Inappropriate activation of ET_A_ receptors is thought important in the pathophysiology of cardiovascular and renal diseases [20–25]. The endothelin system in the kidney and heart was shown to be markedly activated in animals with heart failure [26,27], and its prolonged upregulation proved maladaptive [22,24,25].

Therefore, the upregulated endothelin system might be an important target for therapeutic intervention in heart failure [22,24,25]. Indeed, Sakai *et al.*[28] reported that in heart failure post myocardial infarction long-term ET_A_ blockade improved the survival rate, an analogy to the improvement obtained with angiotensin-converting enzyme (ACE) inhibition which resulted in the introduction of angiotensin-converting enzyme inhibitor (ACEi) as a gold standard therapy of heart failure [29]. However, application of the endothelin system blockade yielded controversial results [21,22,30–32], and the effects in heart failure patients, admittedly receiving nonselective endothelin receptor antagonist (bosentan) or presumably selective ET_A_ antagonist (darusentan) were disappointing: early fluid retention actually leading to worsening of heart failure was a common finding [33–35]. In the landmark ENABLE study (Endothelin Antagonism with Bosentan and Lowering of Events) [36] endothelin receptor antagonist treatment was not recommended in heart failure patients; however, the pertinent experimental studies should continue [37]. Evidently, the effects of genuinely selective ET_A_ receptor blockade on the natural course of heart failure have not yet been evaluated [21–25]. The availability of orally active and indisputably selective ET_A_ receptor antagonist, atrasentan [21], enables exploration of this issue [21,22,25,38–41].

The rat model of volume overload induced by the creation of the aorto-caval fistula (ACF) reasonably well mimics human heart failure [13,15,42–47] and is officially recommended for preclinical studies [48,49]. The Ren-2 renin transgenic rat (TGR) model combines endogenous activation of the RAAS and hypertension [50,51], the two factors critical for the progression of heart failure [18,19,52,53]. We have shown that TGR with ACF exhibited markedly enhanced heart failure-related mortality [15,45,47]. Taking advantage of such suitable experimental research models and the availability of a highly selective ET_A_ receptor antagonist, we evaluated the effects of chronic atrasentan treatment on morbidity and mortality in ACF TGR.

To explore in more detail a possible role of interaction of the RAAS and endothelin system and sympathetic nervous system (SNS) [18,54–56] in the pathophysiology of ACF-induced heart failure, kidney tissue concentrations of angiotensin II (ANG II), ET-1 and norepinephrine were measured. In addition, in critical time-points of the experiments, we assessed the cardiac structure and function, using echocardiography and invasive pressure–volume analysis of the LV.

## METHODS

### Ethical approval, animals, heart failure model, and pharmacological therapeutic regimes

The studies were performed in accordance with guidelines and practices established by the Animal Care and Use Committee of the Institute for Clinical and Experimental Medicine, Prague, which accord with the European Convention on Animal Protection and Guidelines on Research Animal Use and approved by the Ministry of Health of the Czech Republic (project decision 26306/2020-4/OVZ). Heterozygous TGR were generated by breeding male homozygous TGR with female homozygous Hannover Sprague-Dawley (HanSD) rats. Male TGR and HanSD rats, at the initial age of 9 weeks, derived from several litters, were randomly assigned to experimental groups to make sure that the animals from a single litter did not prevail in any group. To obtain reliable data regarding the effects of two treatment regimens on the survival rate, high initial *n* values were used (not so for sham-operated animals) to enable a valid comparison of the long-term survival rate. Such required n values were established using the statistical power analysis method developed by Cohen [57].

Rats were anesthetized (tiletamine + zolazepam, Virbac SA, Carros Cedex, France, 8 mg/kg; and xylasine, Spofa, Czech Republic, 4 mg/kg intramuscularly) and heart failure was induced by volume overload caused by ACF created using needle technique as employed and validated by many investigators, including our own group [13,15,42–47,58,59].

Trandolapril (2 mg/l in drinking water; Gopten; Abbott, Prague, Czech Republic), was used to inhibit ACE because in our previous studies and here in preliminary experiments we demonstrated that at this dose the ACEi, trandolapril, provided maximal blockade of the RAAS and was well tolerated both by rats with ACF-induced heart failure and by sham-operated animals [15,47,59]. ET_A_ receptor blockade was achieved with atrasentan (5 mg/kg per day in drinking water; Abbott, Illinois, USA). The dose of atrasentan was adjusted weekly to actual water intake; such dosage was previously found to effectively block ET_A_ receptors [39,40].

### Detailed experimental design

**FIGURE 1:**
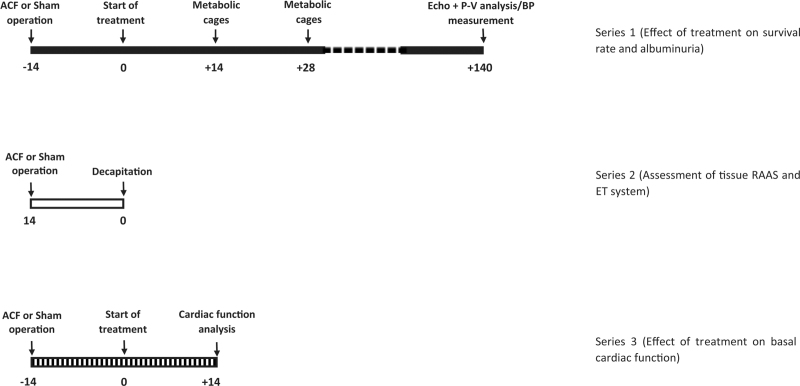
The experimental design of the whole study, delineating the time sequence of experimental maneuvers and different treatment regimes.

The whole experimental design of the study, with a presentation of the detailed time sequence of experimental maneuvers and different treatment regimes, is given in Fig. 1.

### Series 1: Effects of treatment with endothelin type A receptor antagonist and angiotensin-converting enzyme inhibitor, alone or combined, on the survival rate and albuminuria

Animals underwent either sham-operation or ACF creation and were left without treatment for 2 weeks. At this time point (day 0) they were assigned to the following experimental groups:

Group 1: Sham-operated HanSD rats + placebo (initial *n* = 12).
Group 2: Sham-operated TGR + placebo (initial *n* = 14).
Group 3: ACF TGR + placebo (i.e. untreated ACF TGR) (initial *n* = 30).
Group 4: ACF TGR + ET_A_ receptor antagonist (initial *n* = 31).
Group 5: ACF TGR + ACEi (initial *n* = 32).
Group 6: ACF TGR + ACEi + ET_A_ receptor antagonist (initial *n* = 33).

The follow-up period was 20 weeks. At the end of the experiment (on day +140), the survived rats were anesthetized and echocardiography was performed. Subsequently, LV functions were invasively assessed by employing pressure–volume analysis by techniques and protocols developed and validated for mice and rats by Pacher *et al.*[60]. This method was employed almost 10 years ago in our laboratory and it is routinely used in our studies evaluating cardiac functions in rats. Detailed descriptions can be found in numerous of our previous studies [15,47,61,62]. Briefly, rats were anesthetized with long-term anesthesia (thiopental sodium, 50 mg/kg, intraperitoneally, VAUB Pharma a.s., Roztoky, Czech Republic) commonly used for pressure–volume analysis [60]. Before the pressure–volume analysis, echocardiography was performed. Rats were intubated with a plastic cannula to ensure chest relaxation during the whole operation. The left jugular vein was cannulated for securing central venous access for solutions administration as required. A balloon catheter (LeMaitre Single Lumen Embolectomy Catheter, 2F, Burlington, Massachusetts, USA) was inserted under ultrasonic control via the right jugular vein to the vena cava inferior, below the diaphragm to maintain the best position for preload reduction. Just before the pressure–volume measurement of the LV, the conductance and pressure signals of the Millar pressure–volume catheter (Millar, 2F, Houston, Texas, USA) were calibrated using MPVS software (V2.2, Millar) according to the manufacturer's instructions. Functions of the LV were invasively assessed by a pressure–volume catheter introduced into the LV via the right carotid artery as described in previous studies [15,47,61,62]. For basal measurements, pancuronium (1 mg/kg, intravenously, Inresa Arzneimittel, Freiburg, Germany) was administered through the cannulated left jugular vein and rinsed with a bolus of saline to reduce noisiness in the signal caused by breathing. For effective determination of cardiac functions, the preload reductions were performed by slowly inflating the balloon catheter with aqua pour injection. Volume signal was calibrated by end-diastolic and end-systolic volume obtained shortly before invasive recordings. Data from pressure–volume loops were captured and analyzed in LabChart Pro software (ADInstruments, Bella Vista, New South Wales, Australia).

### Series 2: Assessment of angiotensin II, endothelin-1, angiotensin 1–7, and norepinephrine levels and organ weights in the early phase after aorto-caval fistula-induced heart failure

Animals underwent either sham-operation or ACF creation and were left without treatment for 2 weeks and then were killed by decapitation. Whole kidney ANG II, angiotensin 1–7 (ANG 1–7) and norepinephrine levels, and ET-1 concentrations in the kidney cortex, kidney papilla, and lung tissue were measured, as described in our previous studies [38,39,45–47,51,63,64]. The following experimental groups (*n* = 11 each) were investigated:

Group 1: Sham-operated HanSD rats.
Group 2: Sham-operated TGR.
Group 3: ACF TGR.

### Series 3: Effects of 2-week treatment with endothelin type A receptor antagonist and angiotensin-converting enzyme inhibitor, alone or combined, on basal cardiac function assessed by echocardiography and by pressure–volume analysis

Animals were prepared as described in series 1 and 2, and at week 0 the pharmacological treatment was applied for a period of 2 weeks. On day +14, the measurements were performed in the following groups:

Group 1: Sham-operated HanSD rats + placebo (*n* = 7).
Group 2: Sham-operated TGR + placebo (initial *n* = 7).
Group 3: ACF TGR + placebo (i.e., untreated ACF TGR) (*n* = 10).
Group 4: ACF TGR + ET_A_ receptor antagonist (*n* = 9).
Group 5: ACF TGR + ACEi (*n* = 9).
Group 6: ACF TGR + ACEi + ET_A_ receptor antagonist (*n* = 9).

### Statistical analysis

Statistical analysis of the data was performed using Graph-Pad Prism software (Graph Pad Software, San Diego, California, USA). Comparison of survival curves was performed by log-rank (Mantel-Cox) test followed by Gehan-Breslow-Wilcoxon test. Statistical comparison of other results was made by Student's *t* test, Wilcoxon's signed-rank test for unpaired data, or one-way analysis of variance when appropriate. The values are expressed as the means ± standard error of the mean and n represents the number of animals. A *P* value less than 0.05 was considered statistically significant.

## RESULTS

### Effects of treatment with endothelin type A receptor antagonist and angiotensin-converting enzyme inhibitor, alone or combined, on the survival rate and albuminuria

**FIGURE 2:**
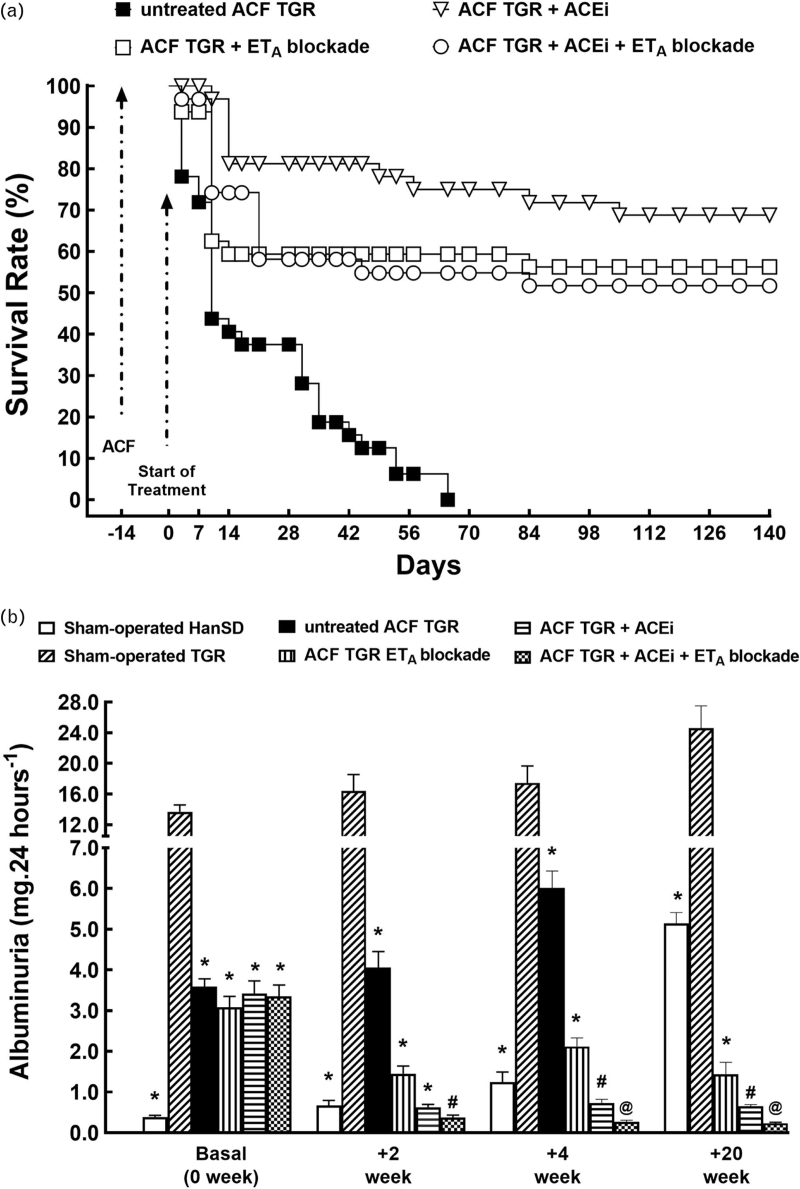
Effects of treatment on survival and albuminuria. The survival rate (a) and albuminuria (b) in sham-operated transgene-negative Hannover Sprague-Dawley rats, sham-operated heterozygous Ren-2 renin transgenic rats, and Ren-2 renin transgenic rats with aorto-caval fistula, treated with endothelin type A receptor antagonist, or with angiotensin-converting enzyme inhibitor, alone or combined. ^**∗**^*P* < 0.05 versus sham-operated Ren-2 renin transgenic rats. ^**∗∗**^*P* < 0.05 versus sham-operated Hannover Sprague-Dawley rats. ^∗∗∗^*P* < 0.05 versus all other groups.

All sham-operated HanSD rats and TGR survived until the end of the study, and for clarity of presentation they are omitted from Fig. 2. As shown in Fig. 2a, untreated ACF TGR definitely began to die from day +14 (4 weeks after the creation of ACF), and by day +65 all the animals were dead. ET_A_ receptor antagonist and ACEi, applied alone, improved survival: the final rate was 56% (18 of 31 animals) and 69% (22 of 32 animals), respectively. With the combined treatment the final survival rate was 52% (17 of 33 animals). The three variants of treatment did not significantly differ in their effectiveness.

At the start of the treatment (2 weeks after sham-operation or creation of ACF), the sham-operated TGR showed about 35-fold higher albuminuria than observed in sham-operated HanSD rats (Fig. 2b). Significantly, the creation of ACF caused a significant about 65% decrease in albuminuria in TGR in this period. Albuminuria modestly but significantly increased throughout the study in sham-operated animals, in parallel with increasing age but, surprisingly, such age-dependent rise was relatively more pronounced in sham-operated HanSD rats. All three treatments reduced albuminuria in ACF TGR, but combined ACE and ET_A_ receptor blockade was the most effective. Remarkably, in ACF TGR receiving the combined treatment albuminuria was even 22-fold lower than in sham-operated HanSD rats (0.236 ± 0.02 vs. 5.14 ± 0.27 mg/24 h, *P* < 0.05).

### Tissue angiotensin II, angiotensin 1–7, norepinephrine, and endothelin-1 levels and organ weights in the early phase after aorto-caval fistula-induced heart failure

Two weeks after the creation of ACF, TGR displayed a further increase in cardiac LV hypertrophy when compared with sham-operated TGR, and marked right ventricle (RV) hypertrophy (Table 1). In addition, ACF TGR displayed substantial lung congestion (increased wet lung weight) without significant differences in body, kidney, and liver weight.

**TABLE 1:** Organ weights 2 weeks after the creation of the aorto-caval fistula or sham-operation, that is, before initiation of treatment protocols (week 0)

	Group		
	HanSD + water	TGR + water	ACF TGR + water
Tibia length (mm)	38.1 ± 0.3	37.9 ± 0.2	37.8 ± 0.3
Whole heart weight (mg)/tibia length (mm)	34.67 ± 0.64	43.54 ± 0.46^∗^	54.26 ± 0.88^∗∗^
LV weight (mg)/tibia length (mm)	24.91 ± 0.17	32.74 ± 0.51^∗^	35.53 ± 0.62^∗∗^
RV weight (mg)/tibia length (mm)	5.93 ± 0.16	6.31 ± 0.29	10.40 ± 0.48^∗∗^
Lung weight (mg)/tibia length (mm)	44.41 ± 0.86	46.72 ± 1.08	78.79 ± 1.14^∗∗^
Kidney weight (mg)/tibia length (mm)	37.01 ± 0.76	38.79 ± 0.69	40.08 ± 1.09
Liver weight (mg)/tibia length (mm)	410 ± 13	420 ± 18	421 ± 16

The values are the means ± SEM. ACF, aorto-caval fistula; HanSD, Hannover Sprague-Dawley rats; LV, left ventricle; RV, right ventricle; TGR, Ren-2 renin transgenic rats.

∗ *P* < 0.05 vs. sham-operated HanSD rats.

∗∗ *P* < 0.05 vs. TGR + water.

**FIGURE 3:**
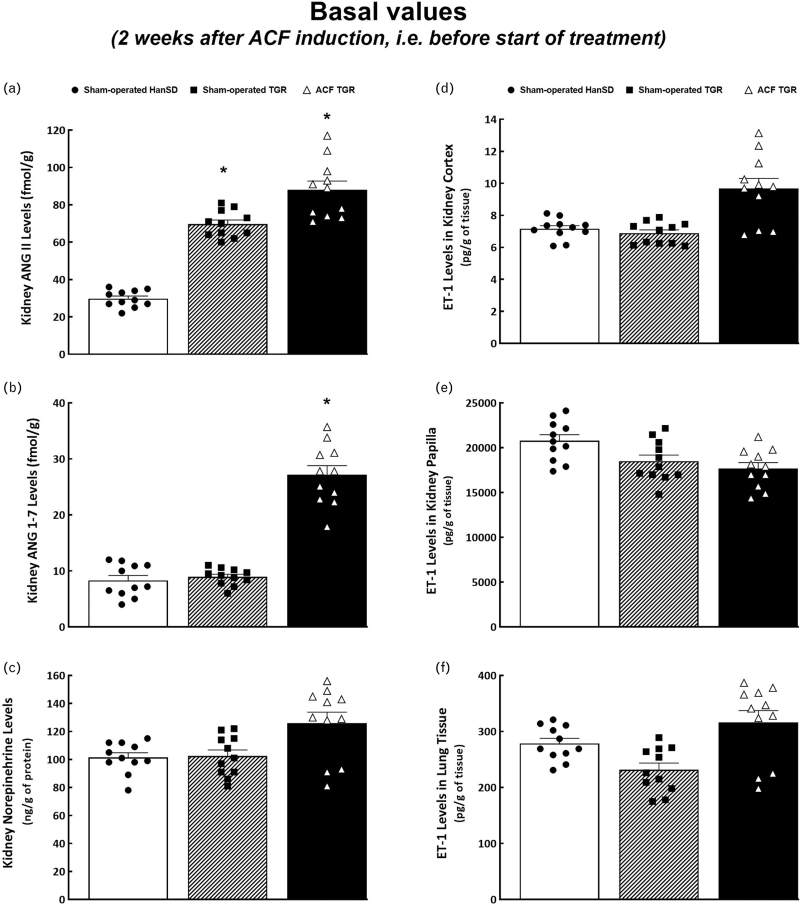
Tissue levels of angiotensin II (a), angiotensin 1–7 (b), norepinephrine (c), endothelin-1 (d) in kidney cortex, endothelin-1 in kidney papilla (e), and endothelin-1 in lung tissue (f) in sham-operated transgene-negative Hannover Sprague-Dawley rats, sham-operated heterozygous Ren-2 renin transgenic rats, and Ren-2 renin transgenic rats with aorto-caval fistula 2 weeks after the creation of the aorto-caval fistula or sham-operation. ^∗^*P* < 0.05 versus sham-operated Hannover Sprague-Dawley rats.

Two weeks after the creation of ACF or sham-operation tissue concentrations of ANG II, ANG 1–7, norepinephrine, and ET-1 were as shown in Fig. 3. Sham-operated TGR showed higher kidney ANG II levels compared with sham-operated HanSD rats (Fig. 3a). Dissimilarly, kidney ANG 1–7 concentrations did not differ (Fig. 3b). Evidently, the intrarenal balance between the vasodilator and vasoconstrictor axes of the RAAS (expressed as the ratio of ANG 1–7 to ANG II) was shifted toward the vasoconstrictor axis. Kidney ANG II levels tended to be higher in ACF TGR (NS), however, the creation of ACF distinctly increased kidney ANG 1–7 levels. This marked increase resulted in a considerable increase in the ANG 1–7/ANG II ratio, up to the level found in sham-operated HanSD rats. There were no significant differences in kidney norepinephrine concentrations between experimental groups (Fig. 3c). Nor were there any significant between-group differences in the concentrations of ET-1 in the kidney cortex, kidney papilla, and lung tissue (Fig. 3d–f).

### Effects of 2-weeks’ treatment with endothelin type A receptor antagonist and angiotensin-converting enzyme inhibitor, alone or combined, on basal cardiac function assessed by echocardiography and by pressure–volume analysis

Sham-operated TGR displayed whole cardiac and LV hypertrophy as compared with sham-operated HanSD rats (Table 2). In TGR the hypertrophy was slightly but significantly greater than observed at week 0 (Table 1). Untreated ACF TGR displayed, again, bilateral cardiac hypertrophy that strikingly progressed over 2 weeks. The final increase above the values from week 0 (see Table 1) was by 27, 16, and 45% in the case of whole cardiac, LV, and RV hypertrophy, respectively. All treatment regimens substantially attenuated the degree of hypertrophy in ACF TGR; the concurrent lung weight decrease suggested attenuation of lung congestion.

**TABLE 2:** Organ weights 4 weeks after the creation of the aorto-caval fistula or sham-operation and after 2 weeks’ treatment with endothelin type A receptor antagonist and angiotensin-converting enzyme inhibitor, alone or combined (week + 2)

	Group					
	HanSD + water	TGR + water	ACF TGR + water	ACF TGR + ACEi	ACF TGR + ET_A_ antagonist	ACF TGR + ACEi + ET_A_ antagonist
Tibia length (mm)	37.9 ± 0.3	38.1 ± 0.2	37.8 ± 0.2	38.1 ± 0.2	38.1 ± 0.3	37.9 ± 0.2
Whole heart weight (mg)/tibia length (mm)	37.21 ± 0.54	48.47 ± 0.98^∗^	69.03 ± 1.18^∗∗^	54.33 ± 1.15^∗∗∗^	47.91 ± 0.75^∗∗∗^	54.79 ± 2.04^∗∗∗^
LV weight (mg)/tibia length (mm)	24.12 ± 0.11	35.12 ± 0.91^∗^	41.09 ± 0.92^∗∗^	33.96 ± 0.68^∗∗∗^	30.81 ± 0.59^∗∗∗^	35.06 ± 1.35^∗∗∗^
RV weight (mg)/tibia length (mm)	7.28 ± 0.19	7.46 ± 0.25	15.09 ± 0.39^∗∗^	12.05 ± 0.17^∗∗∗^	9.92 ± 0.24^∗∗∗^	10.75 ± 0.31^∗∗∗^
Lung weight (mg)/tibia length (mm)	48.96 ± 1.06	48.52 ± 1.29	75.11 ± 1.38^∗∗^	61.91 ± 1.27^∗∗∗^	57.29 ± 1.29^∗∗∗^	61.52 ± 1.33^∗∗∗^
Kidney weight (mg)/tibia length (mm)	40.14 ± 0.41	42.19 ± 1.29	40.08 ± 1.09	38.92 ± 1.36	39.14 ± 1.08	38.99 ± 0.91
Liver weight (mg)/tibia length (mm)	437 ± 19	459 ± 17	445 ± 26	439 ± 26	444 ± 23	433 ± 27

The values are the means ± SEM. ACEi, angiotensin-converting enzyme inhibitor; ACF, aorto-caval fistula; ET_A_, endothelin type A; HanSD, Hannover Sprague-Dawley rats; LV, left ventricle; RV, right ventricle; TGR, Ren-2 renin transgenic rats.

∗ *P* < 0.05 vs. sham-operated HanSD rats.

∗∗ *P* < 0.05 vs. TGR + water.

∗∗∗ *P* < 0.05 vs. ACF TGR + water.

Evaluation of cardiac structure and function by echocardiography showed that sham-operated TGR displayed higher LV anterior and posterior wall thickness and the LV relative wall thickness as compared with sham-operated HanSD rats (Table 3), showing effects of hypertension and LV cardiac hypertrophy. Otherwise, there were no structural and functional LV changes or significant differences between sham-operated TGR and sham-operated HanSD rats. Nor were there any significant differences in RV parameters between sham-operated TGR and sham-operated HanSD rats. Untreated ACF TGR had increased stroke volume and cardiac output (a consequence of the shunt), strikingly increased LV and RV diameters, and decreased LV anterior and posterior wall thickness and LV relative wall thickness (indices of eccentric cardiac hypertrophy). In addition, untreated ACF TGR displayed decreased LV ejection fraction and LV fractional shortening as compared with sham-operated TGR (impairment of LV systolic function). In contrast, at this stage, untreated ACF TGR did not show any impairment of RV systolic function as seen from the normal RV ejection fraction. The treatment with ET_A_ receptor antagonist alone or ACEi alone reduced both the LV and RV diameters but did not change the LV wall thickness and systolic function of the LV in ACF TGR. Notably, combined treatment with ACEi and ET_A_ receptor antagonists did not modify the LV and RV diameters but further decreased the LV anterior and posterior wall thickness and LV relative wall thickness and, unexpectedly, decreased RV ejection fraction.

**TABLE 3:** Echocardiographic analysis performed 4 weeks after the creation of the aorto-caval fistula or sham-operation and after 2 weeks’ treatment with endothelin type A receptor antagonist and angiotensin-converting enzyme inhibitor, alone or combined (week + 2)

	Group					
	HanSD + water	TGR + water	ACF TGR + water	ACF TGR + ACEi	ACF TGR + ET_A_ antagonist	ACF TGR + ACEi + ET_A_ antagonist
Heart rate (s^−1^)	381 ± 16	379 ± 11	369 ± 9	368 ± 8	372 ± 9	377 ± 16
LV diastolic diameter (mm)	6.44 ± 0.12	6.14 ± 0.13	9.89 ± 0.21^∗^	8.74 ± 0.21^∗∗^	8.54 ± 0.23^∗∗^	10.53 ± 0.23^∗^
LV systolic diameter (mm)	3.32 ± 0.12	3. 07 ± 0.12	6.01 ± 0.22^∗^	5.22 ± 0.17^∗∗^	4.97 ± 0.22^∗∗^	6.22 ± 0.22^∗^
LV anterior wall thickness in diastole (mm)	2.04 ± 0.04	2.82 ± 0.04^∗∗∗^	2.11 ± 0.05^∗^	2.06 ± 0.04^∗^	2.01 ± 0.03^∗^	1.66 ± 0.05^∗∗^
LV posterior wall thickness in diastole (mm)	2.18 ± 0.05	3.17 ± 0.08^∗∗∗^	2.29 ± 0.08^∗^	2.29 ± 0.05^∗^	2.15 ± 0.05^∗^	1.86 ± 0.04^∗∗^
LV relative wall thickness	0.62 ± 0.02	1.05 ± 0.06^∗∗∗^	0.42 ± 0.03^∗^	0.44 ± 0.03^∗^	0.51 ± 0.04^∗^	0.35 ± 0.01^∗^
LV ejection fraction (%)	79.2 ± 1.2	80.6 ± 0.9	65.2 ± 0.9^∗^	64.8 ± 1.3^∗^	70.9 ± 1.3^∗^	68.9 ± 1.4^∗^
LV fractional shortening (%)	50.5 ± 1.4	51.5 ± 0.7	38.8 ± 1.1^∗^	39.3 ± 1.1^∗^	42.2 ± 1.2^∗^	41.1 ± 1.5^∗^
LV stroke volume (μl)	149 ± 5.4	146 ± 7.9	384 ± 19^∗^	334 ± 14^∗^	303 ± 14^∗^	373 ± 21^∗^
Cardiac output (ml/min)	61.1 ± 1.5	59.8 ± 3.6	135.7 ± 5.6^∗^	121.5 ± 6.6^∗^	114.6 ± 4.4^∗^	152.9 ± 8.8^∗^
RV diastolic diameter (mm)	3.21 ± 0.07	3.16 ± 0.08	5.71 ± 0.33^∗^	5.02 ± 0.21^∗^	3.94 ± 0.09^∗∗^	5.75 ± 0.22^∗^
RV systolic diameter (mm)	3.05 ± 0.04	2.55 ± 0.11	5.04 ± 0.31^∗^	3.56 ± 0.21^∗∗^	3.49 ± 0.07^∗∗^	5.31 ± 0.19^∗^
RV ejection fraction (%)	57.7 ± 1.7	55.1 ± 2.1	51.1 ± 2.8	50.9 ± 2.4	52.1 ± 2.5	37.7 ± 2.1^∗∗^

The values are the means ± SEM. ACEi, angiotensin-converting enzyme inhibitor; ACF, aorto-caval fistula; ET_A_, endothelin type A; HanSD, Hannover Sprague-Dawley rats; LV, left ventricle; RV, right ventricle; TGR, Ren-2 renin transgenic rats.

∗ *P* < 0.05 vs. TGR + water.

∗∗ *P* < 0.05 vs. ACF TGR + water.

∗∗∗ *P* < 0.05 vs. sham-operated HanSD rats.

**FIGURE 4:**
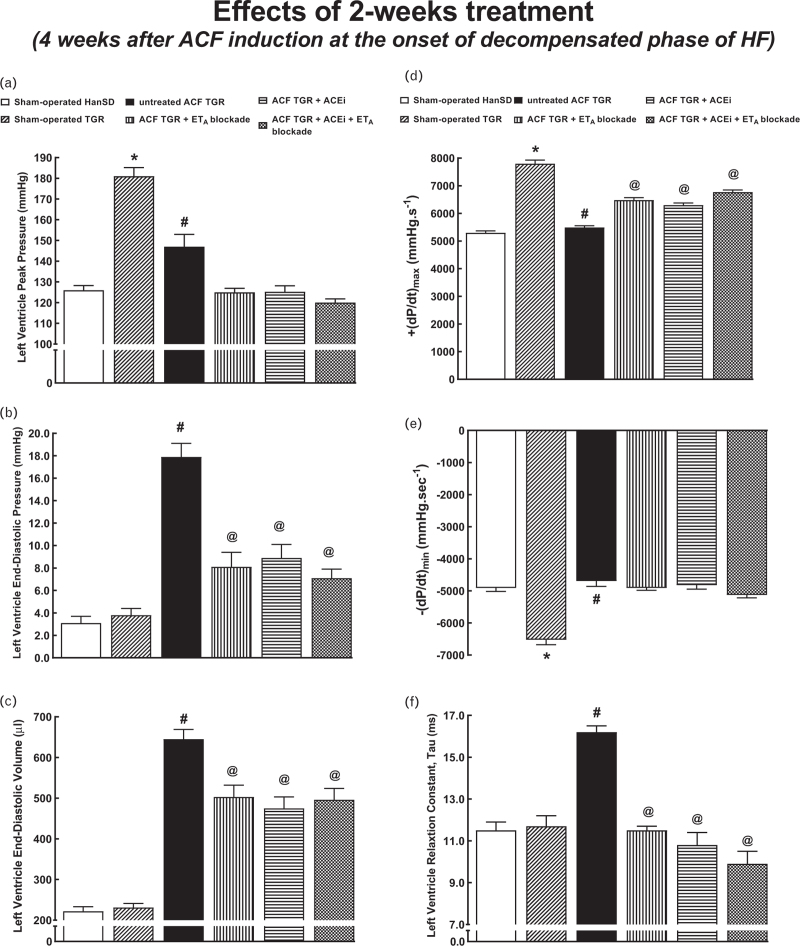
Left ventricle cardiac function assessment by invasive hemodynamic analysis performed 4 weeks after the creation of the aorto-caval fistula, that is, 2 weeks after initiation of treatments in sham-operated transgene-negative Hannover Sprague-Dawley rats, sham-operated heterozygous Ren-2 renin transgenic rats and Ren-2 renin transgenic rats with aorto-caval fistula, treated with either endothelin type A receptor antagonist alone or with angiotensin-converting enzyme inhibitor alone or with the combination of endothelin type A receptor antagonist and angiotensin-converting enzyme inhibitor. Left ventricle peak pressure (a), left ventricle end-diastolic pressure (b), left ventricle end-diastolic volume (c), maximum rates of pressure rise (+dP/dt)_max_ (d), maximum rates of pressure fall (−dP/dt)_max_ (e), left ventricle relaxation constant tau (f). ^**∗**^*P* < 0.05 sham-operated Ren-2 renin transgenic rats versus sham-operated Hannover Sprague-Dawley rats. ^**∗∗**^*P* < 0.05 untreated aorto-caval fistula Ren-2 renin transgenic rats versus sham-operated Ren-2 renin transgenic rats. ^∗∗∗^*P* < 0.05 treated aorto-caval fistula Ren-2 renin transgenic rats versus untreated aorto-caval fistula Ren-2 renin transgenic rats.

**FIGURE 5:**
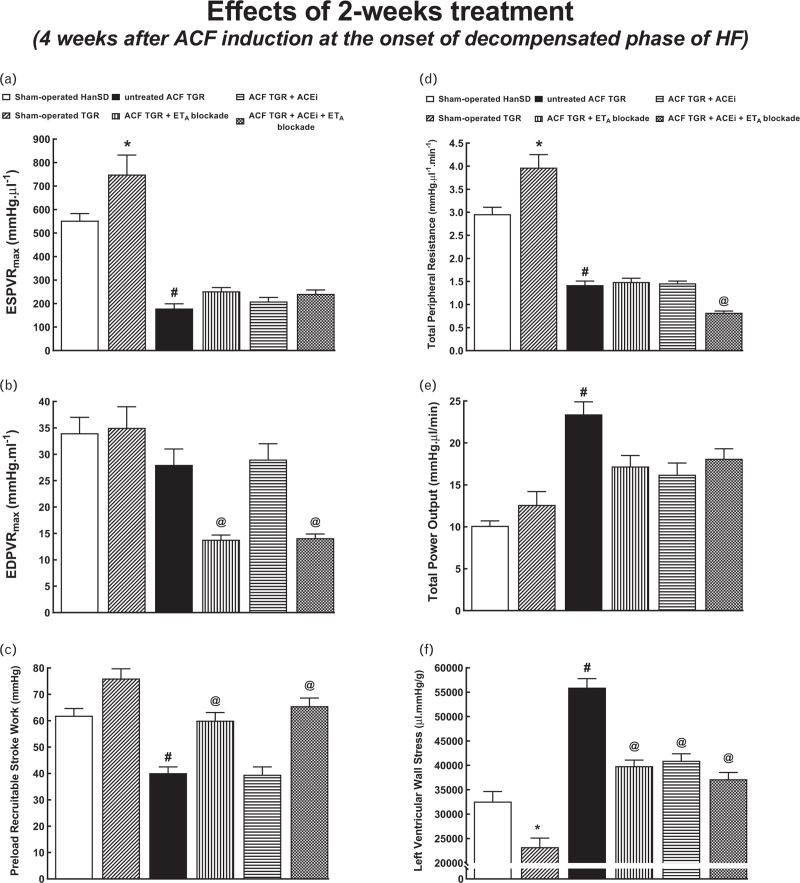
Left ventricle cardiac function assessment by invasive hemodynamic analysis performed 4 weeks after the creation of the aorto-caval fistula, that is, 2 weeks after initiation of treatments in sham-operated transgene-negative Hannover Sprague-Dawley rats, sham-operated heterozygous Ren-2 renin transgenic rats and Ren-2 renin transgenic rats with aorto-caval fistula, treated with either endothelin type A receptor antagonist alone or with angiotensin-converting enzyme inhibitor alone or with the combination of endothelin type A receptor antagonist and angiotensin-converting enzyme inhibitor. End-systolic pressure–volume relationship (a), end-diastolic pressure–volume relationship (b), preload recruitable stroke work (c), total peripheral resistance (d), total power output (e), and left ventricle wall stress (f). ^∗^*P* < 0.05 sham-operated Ren-2 renin transgenic rats versus sham-operated Hannover Sprague-Dawley rats. ^**∗∗**^*P* < 0.05 untreated aorto-caval fistula Ren-2 renin transgenic rats versus sham-operated Ren-2 renin transgenic rats. ^∗∗∗^*P* < 0.05 treated aorto-caval fistula Ren-2 renin transgenic rats versus untreated aorto-caval fistula Ren-2 renin transgenic rats.

On the evaluation of cardiac function by the invasive hemodynamics method (Figs. 4 and 5) sham-operated TGR showed, on one side, higher LV peak pressure (Fig. 4a), maximum rates of pressure rise (+dP/dt)_max_ (Fig. 4d) and end-systolic pressure–volume relationship (ESPVR) (Fig. 5a) and of the total peripheral resistance (TPR) (Fig. 5d) and, on the other side, lower maximum rates of pressure fall (−dP/dt)_max_ (Fig. 4e) and LV wall stress (Fig. 5f) as compared with sham-operated HanSD rats. These results are in line with the degree of hypertension and LV cardiac hypertrophy in sham-operated TGR. Untreated ACF TGR displayed significant decreases in LV peak pressure, (+dP/dt)_max_, (−dP/dt)_max_ (Fig. 4d and e), ESPVR, and preload recruitable stroke work (PRSW) (Fig. 5a and c), and increased LV relaxation constant tau (Fig. 4f) as compared with sham-operated TGR. This indicated impairment of load-dependent as well as load-independent LV systolic function and also of the LV diastolic function. Moreover, untreated ACF TGR showed a marked decrease in TPR (Fig. 5d), and particularly prominent increases in LV end-diastolic pressure (LVEDP), LV end-diastolic volume (LVEDV) (Fig. 4b and c), total power output (Fig. 5e) and LV wall stress (Fig. 5f), as compared with sham-operated TGR. Each of the three treatments decreased LVEDP, LVEDV, LV relaxation constant tau, and LV wall stress and increased (+dP/dt)_max_ in ACF TGR but did not alter (−dP/dt)_max_ and ESPVR. ET_A_ receptor blockade, applied alone or with ACEi, increased PRSW (Fig. 5c) or decreased end-diastolic pressure–volume relationship (Fig. 5b). Finally, only the combined treatment further decreased TPR in ACF TGR (Fig. 5d).

### Effects of 20-weeks’ treatment with endothelin type A receptor antagonist and angiotensin-converting enzyme inhibitor, alone or combined, on organ weights and cardiac function assessed by echocardiography and by pressure–volume analysis

Table 4 collects organ weights from animals that survived until the end of the study (22 weeks after sham-operation or creation of ACF and after 20-weeks’ treatment). The values of whole, LV, and RV weights in the ACF TGR that were treated either with ET_A_ receptor antagonist alone or with ACEi alone were similar with those in untreated ACF TGR in the early phase (4 weeks after the creation of ACF). On the other hand, the extent of lung congestion was lower than observed in untreated ACF TGR (Table 2). Combined treatment with ACEi and ET_A_ receptor antagonist in ACF TGR significantly reduced the bilateral cardiac hypertrophy and lung congestion when compared with ACF TGR treated with ET_A_ receptor antagonist alone or with ACEi alone.

**TABLE 4:** Organ weights determined 22 weeks after the creation of the aorto-caval fistula or sham-operation and after 20-weeks’ treatment with endothelin type A receptor antagonist and angiotensin-converting enzyme inhibitor, alone or combined (week + 20)

	Group				
	HanSD + water	TGR + water	ACF TGR + ACEi	ACF TGR + ET_A_ antagonist	ACF TGR + ACEi + ET_A_ antagonist
Tibia length (mm)	43.6 ± 0.2	43.5 ± 0.3	43.9 ± 0.4	43.6 ± 0.3	43.4 ± 0.5
Whole heart weight (mg)/tibia length (mm)	32.44 ± 0.51	46.52 ± 0.99^∗^	64.95 ± 1.09^∗∗^	68.89 ± 0.53^∗∗^	56.87 ± 0.49^∗∗∗^
LV weight (mg)/tibia length (mm)	26.31 ± 0.21	33.96 ± 0.74^∗^	39.11 ± 0.28^∗∗^	40.37 ± 0.43^∗∗^	34.48 ± 0.32^∗∗∗^
RV weight (mg)/tibia length (mm)	7.44 ± 0.24	7.51 ± 0.18	15.27 ± 0.38^∗∗^	16.58 ± 0.42^∗∗^	12.09 ± 0.21^∗∗∗^
Lung weight (mg)/tibia length (mm)	47.93 ± 1.09	47.66 ± 1.13	63.19 ± 1.21^∗∗^	69.92 ± 2.27^∗∗^	54.19 ± 0.68^∗∗∗^
Kidney weight (mg)/tibia length (mm)	41.89 ± 1.02	41.56 ± 1.22	39.86 ± 1.17	41.89 ± 1.24	40.19 ± 0.97
Liver weight (mg)/tibia length (mm)	451 ± 17	441 ± 23	434 ± 29	438 ± 28	442 ± 25

The values are the means ± SEM. ACEi, angiotensin-converting enzyme inhibitor; ACF, aorto-caval fistula; ET_A_, endothelin type A; HanSD, Hannover Sprague-Dawley rats; LV, left ventricle; RV, right ventricle; TGR, Ren-2 renin transgenic rats.

∗ *P* < 0.05 vs. sham-operated HanSD rats.

∗∗ *P* < 0.05 vs. TGR + water.

∗∗∗ *P* < 0.05 vs. ACF TGR + ACEi and vs. ACF TGR + ET_A_ antagonist.

**FIGURE 6:**
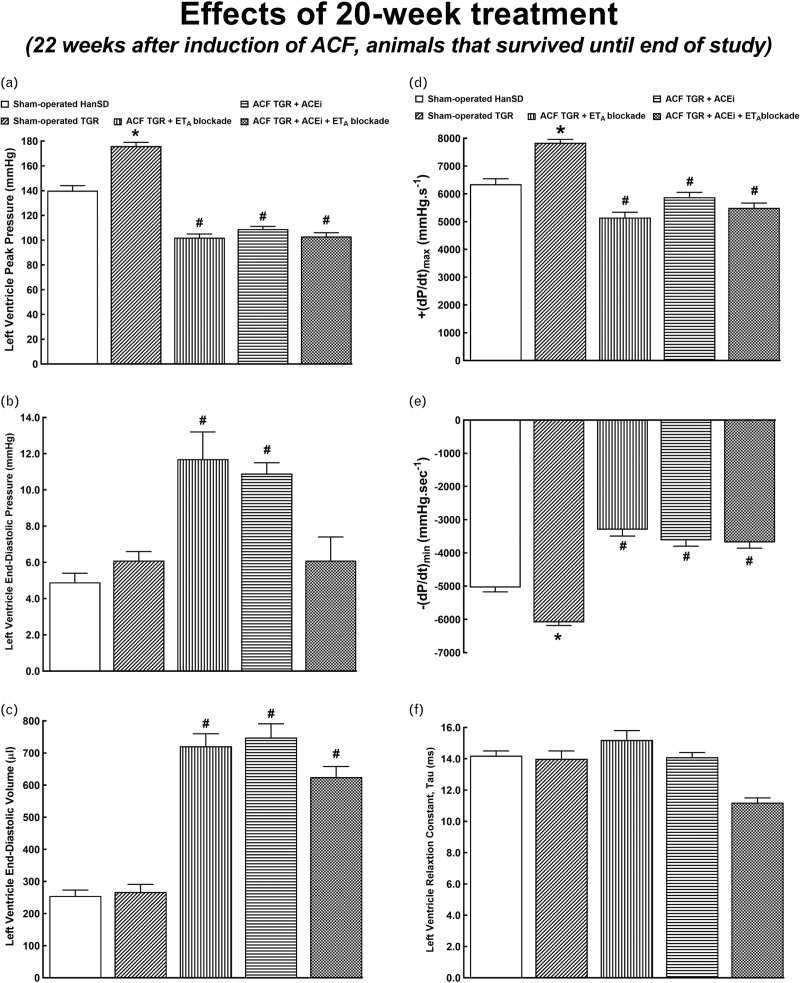
Part 1 of the left ventricle cardiac function assessment by invasive hemodynamic analysis performed 22 weeks after the creation of the aorto-caval fistula, that is, 20 weeks after initiation of treatments in sham-operated transgene-negative Hannover Sprague-Dawley rats, sham-operated heterozygous Ren-2 renin transgenic rats and Ren-2 renin transgenic rats with aorto-caval fistula, treated with either endothelin type A receptor antagonist alone or with angiotensin-converting enzyme inhibitor alone or with the combination of endothelin type A receptor antagonist and angiotensin-converting enzyme inhibitor. Left ventricle peak pressure (a), left ventricle end-diastolic pressure (b), left ventricle end-diastolic volume (c), maximum rates of pressure rise (+dP/dt)_max_ (d), maximum rates of pressure fall (−dP/dt)_max_ (e), left ventricle relaxation constant tau (f). ^∗^*P* < 0.05 sham-operated Ren-2 renin transgenic rats versus sham-operated Hannover Sprague-Dawley rats. ^∗∗∗^*P* < 0.05 treated aorto-caval fistula Ren-2 renin transgenic rats versus sham-operated Ren-2 renin transgenic rats.

**FIGURE 7:**
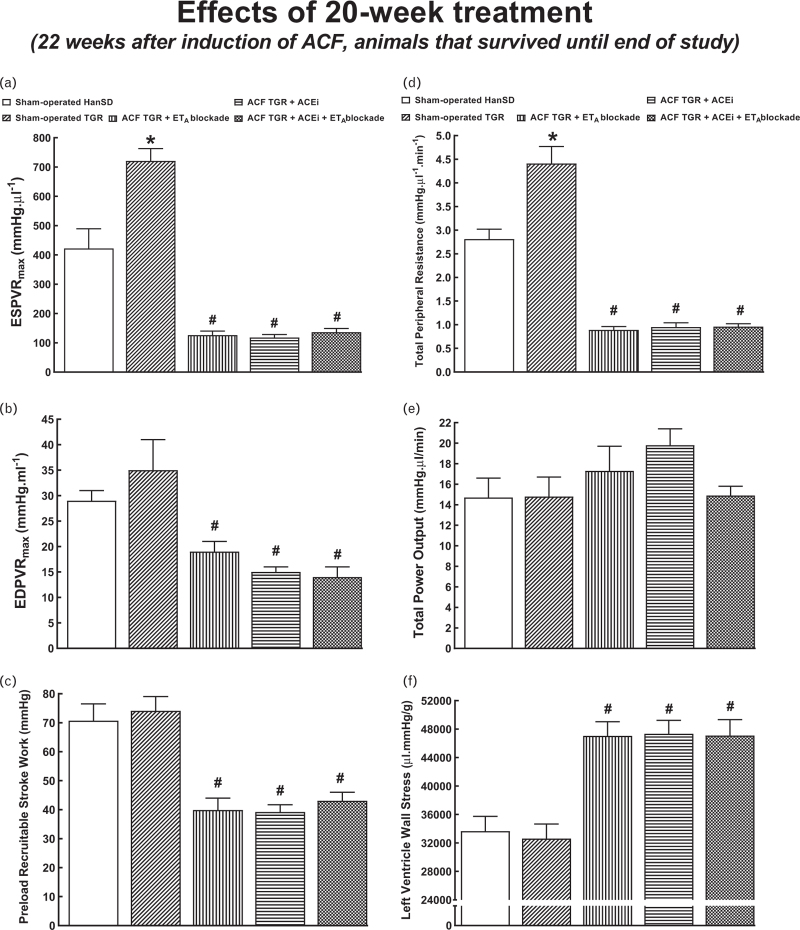
Part 2 of the left ventricle cardiac function assessment by invasive hemodynamic analysis performed 22 weeks after the creation of the aorto-caval fistula, that is, 20 weeks after initiation of treatments in sham-operated transgene-negative Hannover Sprague-Dawley rats, sham-operated heterozygous Ren-2 renin transgenic rats and Ren-2 renin transgenic rats with aorto-caval fistula, treated with either endothelin type A receptor antagonist alone or with angiotensin-converting enzyme inhibitor alone or with the combination of endothelin type A receptor antagonist and angiotensin-converting enzyme inhibitor. End-systolic pressure–volume relationship (a), end-diastolic pressure–volume relationship (b), preload recruitable stroke work (c), total peripheral resistance (d), total power output (e), and left ventricle wall stress (f). ^∗^*P* < 0.05 sham-operated Ren-2 renin transgenic rats versus sham-operated Hannover Sprague-Dawley rats. ^**∗∗**^*P* < 0.05 treated aorto-caval fistula Ren-2 renin transgenic rats versus sham-operated Ren-2 renin transgenic rats.

Table 5 presents an evaluation of cardiac structure and function by echocardiography, again, in the animals that survived until the end of the study. Irrespective of the treatment applied, all ACF TGR groups displayed increases in LV and RV diameters that were even greater than those measured in untreated ACF TGR in the early phase (4 weeks after induction of ACF, see Table 3). In addition, in each of ACF TGR treatment groups, the decreases in LV ejection fraction and LV fractional shortening were more pronounced than those observed in the early phase (see Table 3). Moreover, in contrast to the early phase, all ACF TGR groups exhibited impairment of RV systolic function. Significantly, the treatment regimens that included ET_A_ receptor blockade, alone and combined with ACEi, attenuated the increases in RV diameters and impairment of RV systolic function. Figs. 6 and 7 present an evaluation of cardiac function by the invasive hemodynamics method in animals that survived until the end of the study. All the values for sham-operated HanSD and sham-operated TGR were similar as observed in the early phase (see Figs. 4 and 5). All ACF TGR groups subjected to treatment showed similar load-sensitive values of systolic and diastolic function as well as of load-independent contractile function. Remarkably, the combined treatment with ACEi and ET_A_ receptor antagonist normalized LVEDP in ACF TGR (Fig. 6b) and brought them to levels observed in sham-operated TGR, however, without affecting LVEDV (Fig. 6c). Moreover, there were no significant differences in TPR, total power output and LV wall stress between ACF TGR groups subjected to different treatments (Fig. 7d–f).

**TABLE 5:** Echocardiographic analysis performed 22 weeks after the creation of the aorto-caval fistula or sham-operation, and after 20 weeks’ treatment with endothelin type A receptor antagonist and angiotensin-converting enzyme inhibitor, alone or combined (week + 20)

	Group				
	HanSD + water	TGR + water	ACF TGR + ACEi	ACF TGR + ET_A_ antagonist	ACF TGR + ACEi + ET_A_ antagonist
Heart rate (s^−1^)	376 ± 11	375 ± 11	368 ± 8	353 ± 11	347 ± 16
LV diastolic diameter (mm)	6.91 ± 0.21	7.01 ± 0.21	11.78 ± 0.33^∗^	11.58 ± 0.39^∗^	11.51 ± 0.17^∗^
LV systolic diameter (mm)	3.74 ± 0.18	4.31 ± 0.39	7.77 ± 0.27^∗^	7.85 ± 0.38^∗^	7.69 ± 0.23^∗^
LV anterior wall thickness in diastole (mm)	2.12 ± 0.05	3.08 ± 0.06^∗∗^	2.03 ± 0.05^∗^	2.16 ± 0.09^∗^	1.84 ± 0.07^∗^
LV posterior wall thickness in diastole (mm)	2.51 ± 0.06	3.12 ± 0.09^∗∗^	2.05 ± 0.05^∗^	2.19 ± 0.05^∗^	1.94 ± 0.07^∗^
LV relative wall thickness	0.71 ± 0.04	0.92 ± 0.04^∗∗^	0.35 ± 0.02^∗^	0.40 ± 0.03^∗^	0.37 ± 0.03^∗^
LV ejection fraction (%)	73.6 ± 1.7	72.5 ± 2.1	51.1 ± 1.6^∗^	53.1 ± 2.3^∗^	56.4 ± 1.9^∗^
LV fractional shortening (%)	47.1 ± 1.5	46.1 ± 1.3	30.3 ± 0.7^∗^	32.5 ± 1.5^∗^	32.4 ± 1.3^∗^
LV stroke volume (μl)	182 ± 10	178 ± 9	429 ± 21^∗^	449 ± 31^∗^	404 ± 28^∗^
Cardiac output (ml/min)	64.3 ± 1.3	65.6 ± 3.2	162 ± 5.2^∗^	157 ± 11^∗^	149 ± 12^∗^
RV diastolic diameter (mm)	3.09 ± 0.14	3.06 ± 0.09	6.69 ± 0.21^∗^	5.38 ± 0.43^∗∗∗^	5.61 ± 0.36^∗∗∗^
RV systolic diameter (mm)	2.41 ± 0.11	2.39 ± 0.09	6.05 ± 0.12^∗^	4.57 ± 0.28^∗∗∗^	4.52 ± 0.27^∗∗∗^
RV ejection fraction (%)	67.9 ± 3.9	65.7 ± 3.2	31.9 ± 1.1^∗^	49.3 ± 2.7^∗∗∗^	48.7 ± 2.2^∗∗∗^

The values are the means ± SEM. ACEi, angiotensin-converting enzyme inhibitor; ACF, aorto-caval fistula; ET_A_, endothelin type A; HanSD, Hannover Sprague-Dawley rats; LV, left ventricle; RV, right ventricle; TGR, Ren-2 renin transgenic rats.

∗ *P* < 0.05 vs. TGR + water.

∗∗ *P* < 0.05 vs. sham-operated HanSD rats.

∗∗∗ *P* < 0.05 vs. ACF TGR + ACEi.

## DISCUSSION

We found that both ET_A_ receptor blockade and ACE inhibition substantially reduced the extremely high heart failure-related mortality in ACF TGR. This agrees with the proposal that the blockade of ET_A_ receptors might be an important target for therapeutic intervention in heart failure, at least in its volume-overload variant. A similar favorable action of ACEi (improvement of survival, attenuation of albuminuria, etc.) indicates that even though early activation of the vasoconstrictor/sodium retaining axis of the RAAS may be beneficial, its long-term pleiotropic actions are detrimental and contribute to the progression of heart failure.

Since both treatment regimens substantially delayed the heart failure-related morbidity and mortality in ACF TGR, we hypothesized that combined treatment with trandolapril and atrasentan should provide additive protective actions. However, this hypothesis has not been corroborated: the results of the combined therapy did not significantly differ from those of either single treatment. Therefore, one should consider individual aspects of heart failure-related morbidity as related to interaction at various levels of the RAAS and endothelin systems [65,66]. ANG II can stimulate ET-1 release by various cell types [67–69] and vice versa, ET-1 stimulates ANG II formation [65,70]. Indeed, in TGR after 5/6 renal ablation (5/6 NX), RAAS inhibition decreased ET-1 levels similarly as observed in animals treated with ET_A_ receptor antagonist [38]. Evidently, some of the beneficial effects of the blockade of one system can also be partially mediated, indirectly, by inhibition of the other system.

Notably, the combined treatment exhibited more pronounced effect on albuminuria and was more effective in reducing bilateral cardiac hypertrophy and lung congestion. Since albuminuria and cardiac hypertrophy are independent risk factors for cardiovascular morbidity and mortality (including heart failure-related mortality) [3,7,10,64,71–73], greater beneficial effects of the combined treatment might provide additional protection. To make this conclusive, studies using long-term treatment protocols (e.g. 40 weeks) are required. Nevertheless, the present results strongly suggest a benefit of dual inhibition of the RAAS and ET_A_ system in chronic kidney disease, heart failure, and similar disorders. If so, understanding the underlying mechanism(s) is important.

To address this issue, we examined the effects of the three treatments (atrasentan, trandolapril, or both combined) on cardiac function after 2-weeks’ treatment, at a time when untreated animals were showing high mortality. Untreated ACF TGR not only exhibited bilateral cardiac hypertrophy and prominent eccentric chamber remodeling but also an impairment of load-sensitive systolic and diastolic function and load-independent contractile function of the LV, which confirmed the view that the ACF-induced model of heart failure well represents heart failure with reduced ejection fraction elicited by chronic volume-overload insult [48,49,74].

We hypothesized that the beneficial effects of the treatment regimens on long-term survival were preferentially mediated by cardiac mechanisms, in agreement with the recent evidence that 15 weeks’ ACEi treatment significantly improved LV ejection fraction in ACF TGR [59]. Here we found that ACF TGR which survived until the end of the study and had been exposed to 20 weeks’ treatment exhibited impaired load-dependent as well as load-independent LV contractility. Moreover, irrespective of the treatment variant actually applied, they exhibited markedly increased LV wall stress, bilateral cardiac hypertrophy, and lung congestion. This could reflect a complete failure of our treatment regimens to improve cardiac morphology and function. However, it should be noticed that the data cannot be compared with those obtained at the same time from untreated ACF TGR because all of the latter died 10 weeks earlier. However, if we used for the comparison of the untreated ACF TGR in the early phase of heart failure (Table 2), we find that whole heart, LV, and RV weights and lung weights in the groups treated with either inhibitor for 20 weeks (see Table 4) are similar as in untreated ACF TGR, and with the combined treatment they are even lower. Apparently, the eccentric cardiac remodeling and cardiac hypertrophy related to the enhanced cardiac output (blood recirculation through the fistula) progressed during the study. This was also supported by the data on cardiac function obtained by pressure–volume analysis: the respective values for ACF TGR treated for 20 weeks were similar to those found in untreated ACF TGR in the early phase after ACF-induced heart failure. Furthermore, the combined treatment reduced LVEDP to levels observed in sham-operated TGR.

Overall, each of the treatment regimens applied within the long-term protocol (20 weeks) improved cardiac morphology, systolic and diastolic function of the LV and reduced lung congestion. Most probably, the beneficial effects on the survival rate observed with atresan or trandolapril, alone or combined, are mostly mediated by cardiac mechanisms. This notion is further supported by the effectiveness of 2-weeks’ treatment on cardiac morphology and function. At the onset of heart failure decompensation (when untreated rats were beginning to die) all treatment regimens substantially attenuated bilateral cardiac hypertrophy and lung congestion, reduced LVEDP, LVEDV, LV wall stress, and improved LV systolic contractility.

What was the degree of activation of the intrarenal neurohormonal systems in the earliest phase of ACF-induced heart failure? Two weeks after ACF creation, just before the treatment was initiated, ACF TGR did not display any significant increase in kidney ANG II levels. This suggests no substantial activation of the intrarenal vasoconstrictor/sodium retaining axis of the RAAS. In contrast, elevated kidney ANG 1–7 and increased ANG 1–7/ANG II ratio indicates activation of the intrarenal vasodilator/natriuretic axis of the RAAS. There was no increase in kidney norepinephrine concentration, that is, an absence of substantial activation of the intrarenal SNS. In addition, ACF TGR did not show increased concentrations of ET-1 in the renal cortex, papilla, and lung tissue, which suggests no substantial activation of the tissue endothelin system. Evidently, in the very early phase of volume-overload heart failure, ACF TGR did not show intrarenal activation of the vasoconstrictor/sodium retaining axis of the RAAS, SNS, or endothelin system. However, there was a marked activation of the intrarenal vasodilator/natriuretic axis of the RAAS.

The evidence that kidney activity of RAAS, SNS, and endothelin system is not increased in ACF TGR seems incompatible with the neurohormonal theory of the pathophysiology of heart failure, which proposes that in heart failure the activity of the RAAS and SNS is increased and compensates for the initial insult, even though in the long run such hyperactivity is known to be deleterious and critically contributes to the progression of heart failure [17–19,54–56]. In addition, the present results are at variance with our earlier report on an increased plasma and kidney RAAS and SNS activity in hypertensive rats [15,59,75]. However, this was so 5 weeks after the creation of ACF, in the phase of advanced heart failure decompensation, with 60% mortality (Fig. 5). All this accords with the neurohormonal concept that during the progression of heart failure the neurohormonal activity does increase and counteracts the cardiac function impairment (yet in the long run such inappropriate activation becomes extremely deleterious [17–19,54–56,76]). Briefly, in the very early phase of high-output heart failure, the sodium retaining activity of the RAAS is not apparent. However, in our ACF TGR, kidney ANG 1–7 and the ANG 1–7/ANG II ratio was increased. Evidently, the ACE2/ANG 1–7/Mas receptor axis of the RAAS, which counteracts the effects of the classical RAAS axis [77], was substantially activated. Taken together, since ANG 1–7 is the most important component of the ACE2/ANG 1–7/Mas axis, its activation was presumably the first compensatory event in response to ACF creation, preceding the activation of the vasoconstrictor/sodium retaining axis of the RAAS, SNS, and endothelin system. We believe that this counteracted the subsequently increased activity of the RAAS, SNS, and endothelin systems and attenuated their long-term deleterious influence, in agreement with the proposed role of ANG 1–7, particularly under conditions of elevated kidney ANG II levels [77,78], and with the recent evidence that the elevated ANG 1–7/ANG II ratio predicts a beneficial outcome of heart failure [79].

### Limitations of the study

One limitation of the current study is the application of the ACF TGR model. On the other hand, its major advantage is that the rats are highly hypertensive and display marked systemic and intrarenal activation of the RAAS [50,51], thus they exhibit two most important risk factors for the progression of heart failure [18,19,52,53]. However, the model is sometimes regarded ‘nonnatural’ and the progression of heart failure is thought excessively accelerated. Moreover, the creation of the ACF is associated with a profound decrease in total peripheral resistance and a subsequent decrease in blood pressure: evidently, the initial increase in cardiac output cannot compensate the decrease in TPR. It must also be admitted that the ACF TGR model mimics heart failure dependent on chronic volume overload, a condition affecting only 5–7% of heart failure patients. Most of them suffer from severe mitral insufficiency which is resistant to treatment [3].

It is also admitted that all our series which evaluated cardiac functions were performed in long-term anesthetized and surgically stressed animals, which must have caused the increased activity of neurohormonal systems, particularly of the RAAS and SNS. Possibly, the resultant changes in neurohumoral and volume status could alter cardiac function to some extent, both in healthy animals and in those with ACF-induced heart failure. However, we demonstrated previously, in the initial comprehensive reference study and in our previous studies [15,47,61,62] that long-term anesthesia (isoflurane or barbiturates) and surgical procedures do not deteriorate the stability of animals. Therefore, despite such potential drawbacks the pressure–volume analyses are now accepted as a golden standard approach for the evaluation of cardiac function in mice and rats.

In conclusion, our results showed that, first, even in the absence of pronounced activation of the tissue endothelin system in the early phase of volume-overload heart failure, ET_A_ receptor blockade delays the onset of heart failure decompensation and improves the survival in ACF TGR. Second, the beneficial effects of each of the treatment regimens on long-term survival are most probably mediated by partial recovery of cardiac function, specifically by attenuation of bilateral cardiac hypertrophy, of lung congestion, by reducing LVEDP, LVEDV, LV wall stress, and also by improving LV systolic contractility. Third, the addition of ET_A_ receptor blockade did not increase the protection against heart failure-related mortality in ACF TGR beyond the improvement obtained with the treatment with ACEi alone.

On the whole, our results suggest that targeting the endothelin system should again be considered for the treatment of heart failure, at least of its volume-overload variant, and in individuals with background hypertension and enhanced RAAS activity.

## ACKNOWLEDGEMENTS

Previous presentations: none.

The current study was supported by the project National Institute for Research of Metabolic and Cardiovascular Diseases (Program EXCELES, Project No. LX22NPO5104) – funded by the European Union – Next Generation EU.

The current study was also supported by the Ministry of Health of the Czech Republic grant number 20-02-00052 awarded to H.M. and P.K. was supported by the Grant Agency of Charles University, grant number 68121.

### Conflicts of interest

There are no conflicts of interest.
